# Identification and clinical validation of key genes as the potential biomarkers in colorectal adenoma

**DOI:** 10.1186/s12885-022-10422-9

**Published:** 2023-01-11

**Authors:** Bangting Wang, Jiting Zhang, Xin Wang, Lili Zhao, Yan Wang, Zhining Fan, Li Liu, Wenqing Gao

**Affiliations:** 1 Digestive Endoscopy Department, The First Affiliated Hospital With Nanjing Medical University and Jiangsu Province Hospital, Nanjing, Jiangsu China; 2 State Key Laboratory of Genetic Engineering, School of Life Sciences, Fudan University, Shanghai, China

**Keywords:** Colorectal adenoma, Prognostic signature, GEO, Bioinformatics, Immunity

## Abstract

**Background:**

Colorectal cancer (CRC), ranking third in cancer prevalence and second in mortality worldwide, is mainly derived from colorectal adenoma (CRA). CRA is a common benign disease in the intestine with rapidly increasing incidence and malignant potential. Therefore, this study aimed to recognize significant biomarkers and original pathogenesis in CRA.

**Methods:**

Transcriptome data of GSE8671, GSE37364, and GSE15960 were downloaded from the Gene Expression Omnibus (GEO) datasets, and differentially expressed genes (DEGs) were screened. Functional pathways enrichment, protein–protein interaction (PPI) network, stem-correlation analysis, CIBERSORT, risk score and survival analyses were performed. RT-qPCR and immunohistochemical staining were applied to verify our results.

**Results:**

Screening for significant DEGs in each dataset, we identified 230 robust DEGs, including 127 upregulated and 103 downregulated genes. Functional pathways enrichment showed that these DEGs were distinctly enriched in various tumor-associated pathways, such as growth factor activity, extracellular structure organization, neutrophil activation, and inflammatory response. We filtered out two hub genes via STRING and Modules analysis, including CA2 and HSD11B2. Stem-correlation analysis displayed that hub genes were negatively associated with stem-related genes (Olfm4, CD44, CCND1 and MYC). The CIBERSORT algorithm indicated that Macrophage2, activated mast cells, and Neutrophils promoted CRA progression through inflammation. Survival analysis showed that CA2 and HSD11B2 were positively associated with survival outcomes in CRC.

**Conclusion:**

Our study has successfully identified the critical role of two core genes in the development and oncogenesis of CRA, which provides novel insight into the underlying pathogenesis, potential biomarkers and therapeutic targets.

**Supplementary Information:**

The online version contains supplementary material available at 10.1186/s12885-022-10422-9.

## Introduction

Colorectal cancer (CRC) is a malignant tumor in the intestine with a greatly high incidence, ranking second in male malignant tumors and third in females [1]. In the United States, approximatedly 150,000 new cases of CRC occurred in 2021, with about 53,000 deaths [2]. As developing countries progress, the incidence of colon cancer is gradually increasing globally, and new cases are expected to increase to 2.5 million by 2035 [3]. With changes in diet and lifestyle, the incidence of CRC in China has also increased rapidly, accounting for over 40% of global morbidity in 2020 [4]. The latest studies report that CRC incidence in people under 50 has increased significantly [5], and CRC treatment has greatly burdened patients and society. CRC is mainly sporadic, and about 85% of colorectal cancers originate from colorectal adenoma (CRA), of which 80% of adenomas have APC mutations, on which they accumulate multiple gene mutations (KRAS, p53, and SMAD4) and gradually evolve into cancer [6, 7]. The sequence of colorectal adenoma-cancer evolution usually takes 5–15 years [8], creating an optimal window period for the clinical prevention and treatment of CRA and CRC. Therefore, prevention of CRA and timely and effective blocking of adenoma-cancer sequences are crucial to reducing CRC incidence.

CRA is a benign lesion derived from the glandular epithelium of the colorectum. Pathological classification is divided into three categories, including tubular adenoma, villous adenoma, and villous tubular adenoma. Besides, adenomas can be classified into low-risk and high-risk adenomas based on their malignant potential. High-risk adenomas include histopathologic diagnosis of villous or tubular villous adenomas, ≥ 10 mm in diameter, with or without dysplasia, which is considered as a precancerous lesion of CRC. Epidemiological studies have shown that about 10% of adenomatous polyps will develop colorectal cancer, and about 25% of high-grade adenomas will develop colorectal cancer [9]. CRA incidence is obviously increasing due to different risk factors, such as genetics, age, BMI, alcohol and exercise [10, 11]. Colonoscopy screening and endoscopic adenoma eradication are currently the most effective methods for detecting and treating adenomas, but adenomas are prone to recurrence after resection. Domestic studies have shown that the recurrence rate of CRA can reach 61.09% within two year, and the recurrence rate gradually increases with time [12]. High-risk factors for postoperative recurrence of adenoma include fragment resection, intraoperative bleeding, high-grade adenoma, and lesion size ≥ 40 mm [13]. In addition, after adenoma treatment, according to its specific pathological classification, clinical guidelines recommend repeat colonoscopy every 1–3 years. Lengthy bowel preparations, unbearable pain associated with colonoscopy, intraoperative perforation, and surgical risks of postoperative wound bleeding and infection significantly reduce the patients’ enthusiasm for examination and treatment. Therefore, finding essential genes, exploring their potential pathogenesis of colorectal adenoma, and developing gene-targeted drugs are urgent clinical and scientific problems to be solved.

Herein, we systematically analyzed transcriptomic characteristics of adenoma based on GEO datasets. DEGs (differential expression genes) among colorectal mucosa and adenoma were analyzed and obtained, followed by GO/KEGG, GSEA, and protein–protein interaction (PPI) network analysis. We also exhibited the immune landscape of CRA to the mucosa. Finally, we identified two core genes, CA2 and HSD11B2, confirming their expression patterns through real-time qPCR and immumo-histochemical staining, and verifying the survival rates of each essential gene. In conclusion, our study will improve the understanding of the pathogenesis in CRA, and the core genes may serve as original biomarkers and therapeutic targets for colorectal adenoma and cancer.

## Materials and methods

### Collection of data from GEO datasets

The RNA expression profiles of CRAs were obtained from GEO datasets (http://www.ncbi.nlm.nih.gov/geo/), including GSE8671 [14], GSE15960 [15], and GSE37364 [16], based on the GPL570 platform. Specifically, our study contained 140 samples, including 65 CRAs and 75 mucosal samples (Table 1). Their RNA-sequencing data were processed for identifying DEGs. Datasets that met the following criteria were eligible:

1. CRA and mucosa;
2. datasets contained the transcriptome data from CRA and mucosa;
3. more than 5 pairs of samples among each dataset.

**Table 1:** Details for GEO colorectal mucosa and adenoma tissue data

Dataset	Platform	Samples(mucosa)	Samples(adenoma)
GSE8671	GPL570	31	31
GSE15960	GPL570	6	6
GSE37364	GPL570	38	28

The quantile normalization method normalizes gene expression intensities.

### Identification of DEGs from GEO datasets

The “edgedR” package was applied to identify DEGs in each GEO dataset (GSE8671, GSE15960, and GSE37364) in R (version 4.1.1). These genes satisfied |log2 fold-change (FC)|> 1.00, *p*-adjust < 0.05 and *p-*value < 0.05 were taken into consideration for further statistical analysis. Heatmaps are performed online through the Morphus (https://software.broadinstitute.org/morpheus/). Draw Venn diagrams via Bioinformatics & Evolutionary Genomics (http://bioinformatics.psb.ugent.be/webtools/Venn/).

### GO and KEGG pathway analysis

GO analysis explains the biological function of specific genes from three parts, involed in a cellular component, molecular function, and biological process. KEGG [17, 18], analyzing the role of genes, was employed to find which biological pathways specific genes were enriched in CRAs. Performing GO and KEGG pathway analysis based on DEGs between adenoma and mucosa from GEO datasets via the R package (clusterProfiler, 3.14.3 version).

### Gene Set Enrichment Analysis (GSEA)

RNA-sequence expression of mucosa and adenoma was analyzed by GSEA (http://software.broadinstitute.org/gsea/ version 4.0); clusterProfiler, 3.14.3 version.) referring to h.all.v7.2.symbols.gmt [Hallmarks] and the obtained gene sets were compared with known disease-associated gene sets to characterize CRAs. GSEA analysis was based on the normalized data among GSE8671, GSE15960 and GSE37364.

### Establishment of PPI network

We applied the STRING (https://string-db.org) online database to build the PPI network based on the common DEGs. After obtaining the primary PPI network, we performed Cytoscape (version 3.8.2) software to visualize and analyze the gene interaction network. Use Cytoscape software's MCODE plug-in to identify the basic modules of the entire network, including upregulated and downregulated genes.

### Tissues

Ten pairs of fresh adenoma and adjacent mucosa tissues were collected from the Endoscopy Center of Nanjing Madical University Jiangsu province hospital. Besides, pathological sections of 10 cases of paraffin-embedded tissues (mucosa and adenoma) were obtained from the Department of digestive endoscopy, Nanjing Madical University Jiangsu province hospital too. Ethical approval was obtained for this study from the Ethical Committee of Medical Research, Jiangsu province Hospital of Nanjing Medical University (2018-SR-258).

### Validation of mRNA and protein expressions of hub genes

To confirm the expression of CA2 and HSD11B2, we performed RT-qPCR, the immunohistochemical staining (IHC) for CRAs and normal tissues. We utilized the RNeasy Protect Mini kit (Tiangen) to extract total RNA of mucosal tissues and CRAs. The reverse transcription reagent purchased from Promega was applied to reverse the transcription of RNA. All experimental procedures followed the instructions of the kit. SYBR Green Master mix (Vazyme) was used for polymerase chain reaction (PCR). The workflow includes initial denaturing (15 min at 95 °C), 40 cycles of 95 °C for 30 s and 60 °C for 1 min in the 7500 fast real-time PCR system (BioRad). Primers for RT-qPCR were recited in Supplementary Table S1. 5 μm slides of CRA and normal mucosa were orderly dewaxed in xylene baths for 3 times, then rehydrated with graded alcohol series and retrieved in a pressure cooker with sodium citrate buffer (pH 6.0) heating for 10 min. The CA2 (GeneTex, GTX105562) and HSD11B2 (Proteintech, 14,192–1-AP) antibodies were utilized for IHC according to their protocols. The IHC steps followed our previous article [19]. We took two field shots on each slide. The IHC staining scores (IS) were classified into four score ranks: 0, negative; 1, weak; 2, moderate; and 3, strong. The percentage of positively stained cells (PS): 0 (< 5%), 1 (5–25%), 2 (25–50%), 3 (50–75%) and 4 (75–100%). The score of each slide: IS x PS (0—12) [20].

### Analysis of ROC and AUC

MedCalc software was utilized for ROC analysis of CA2 and HSD11B2 based on RT-qPCR results and GEO datasets (GSE71187 and GSE41657). AUC and ROC were applied to assess the predictive value of the hub genes for CRA and CRC.

### Relationship between gene expression and immune cell infiltration

The CIBERSORT (Cell-type Identification by Estimating Relative Subsets Of RNA Transcripts) was utilized to research the association between colorectal tissues ( mucosa and adenoma) and 22 immune cells. At the same time, we analyzed the association between the expression of core genes (CA2 and HSD11B2) and tumor-infiltrating immune cells via R.

### GEPIA database analysis

The GEPIA database (http://gepia2.cancer-pku.cn/#index) was used to explore the relationship between the gene expression and prognosis in different tumors based on TCGA datasets. In this study, we applied GEPIA to analyze the relationship between expression and prognosis of CA2 and HSD11B2 according to 272 cases of CRC. Besides, the correlation between targeted gene expression and tumor stage was also determined by the GEPIA database. The univariate Cox regression analysis was performed to establish the risk score of hub genes-related prognostic signature based on the TCGA cohort. For the survival analysis, the patients were split medially (50% high-expression and 50% low-expression), and adding the 95% CI as a dotted line.

### Statistical analysis

All the experimental analysis results were shown as means ± the standard deviation (SD). The differences between various groups were analyzed by Graphpad Prism 8. The *t*-test analysis of variance was utilized to evaluate the differences between the two groups. *P-*value < 0.05 was considered to be statistical significance. All bioinformatic analysis was performed via R (V 4.1.1) software.

## Results

### Identification of DEGs in CRA

**Fig. 1:**
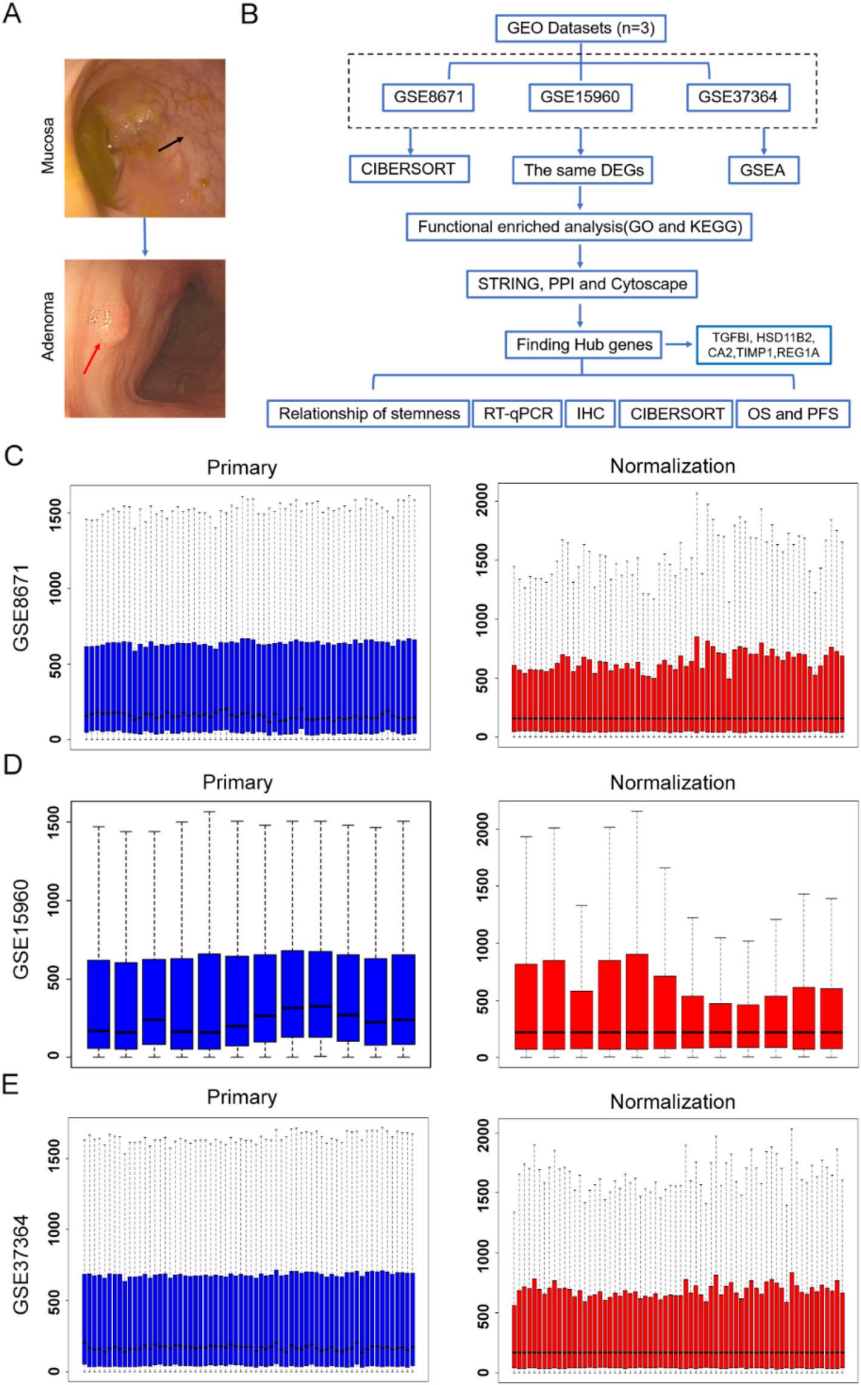
The flow chart of screening hub genes. **A** The evolution of colorectal mucosa into adenoma. **B** Bioinformatic analysis and clinical validation of screening GEO datasets. **C-E** Raw expression and normalized expression data from GSE8671, GSE37364 and GSE15960

**Fig. 2:**
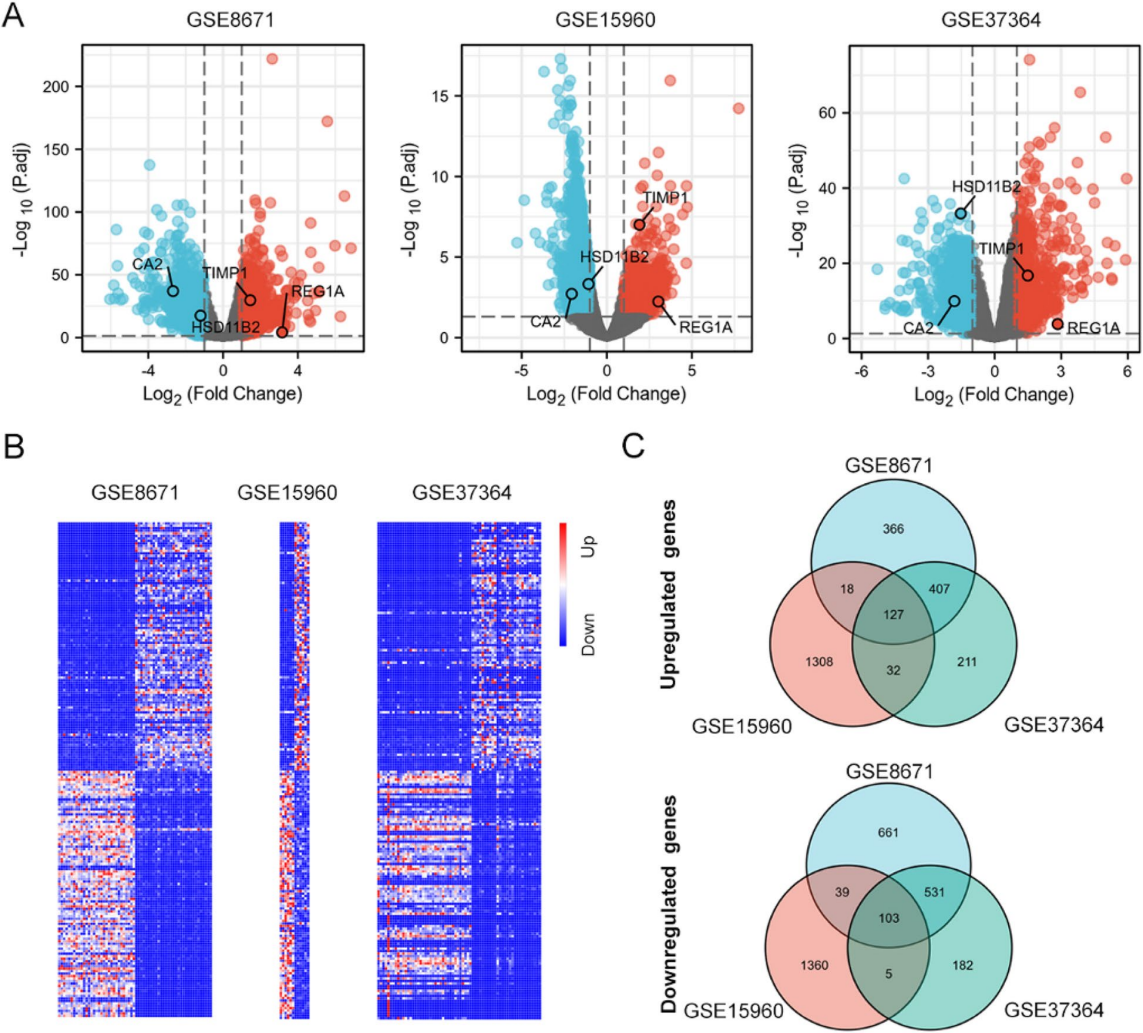
DEGs dentification among three GEO datasets. **A** Volcano plots of DEGs in the three datasets (GEO8671, GEO15960 and GEO37364). There were 2252 DEGs in GSE8671, including 918 upregulated and 1334 downregulated genes; 2992 DEGs in GSE15960, including 1487 upregulated and 1505 downregulated genes; 1598 DEGs in GSE37364, including 777 upregulated and 821 downregulated genes. Red, upregulation; blue, downregulation; grey, none significance. **B** Heatmap of the top 200 DEGs (100 up and 100 down-regulated genes) among the datasets. **C** Venn diagrams of DEGs among the three GEO datasets with the same trends. The overlap included 127 upregulated and 103 downregulated genes

The development of almost CRC follows the mucosa-adenoma-cancer sequence. In order to investigate the significant biological functions of critical DEGs in the evolution of CRA (Fig. 1A), we have performed bioinformatics analysis (GO, KEGG, PPI, and CIBERSORT) in depth (Fig. 1B). First, we selected and downloaded three databases on CRA from GEO, including GSE8671, GSE15960, and GAE37364. 75 CRAs and 65 mucosal tissues were enrolled in this study (Table 1). The three GEO datasets were normalized, and the results are shown in Fig. 1C-E. The volcano plot of each data set was constructed (Fig. 2A), indicating the difference in molecular expression profiles between CRA and mucosa. The DEGs also were displayed by heatmaps in Fig. 2B. There were 2252 DEGs in GSE8671, including 918 upregulated and 1334 downregulated genes; 2992 DEGs in GSE15960, including 1487 upregulated and 1505 downregulated genes; 1598 DEGs in GSE37364, including 777 upregulated and 821downregulated genes. Through the Venn diagram, we identified 127 upregulated genes and 103 downregulated genes in common among the three datasets (Fig. 2C).

### Functional enrichment analysis based on the identical DEGs

**Fig. 3:**
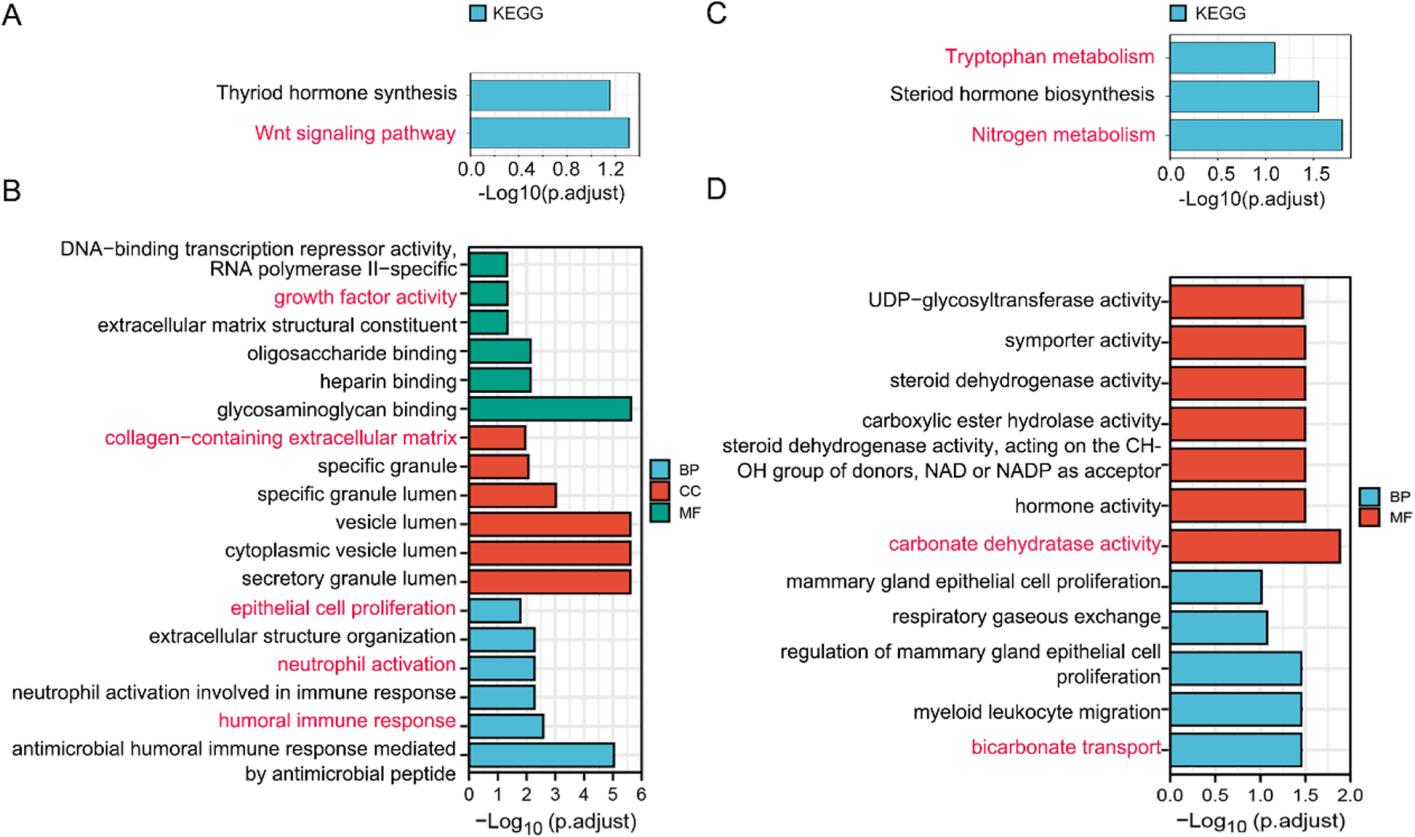
GO and KEGG pathway enrichment analysis. **A** KEGG pathway enrichment analysis of upregulated DEGs. **B** The top 18 significant GO terms of upregulated DEGs. **C** KEGG pathway enrichment analysis of downregulated DEGs. **D** The top significant GO terms of downregulated DEGs. Red, CRA-associated KEGG pathways or GO terms

**Fig. 4:**
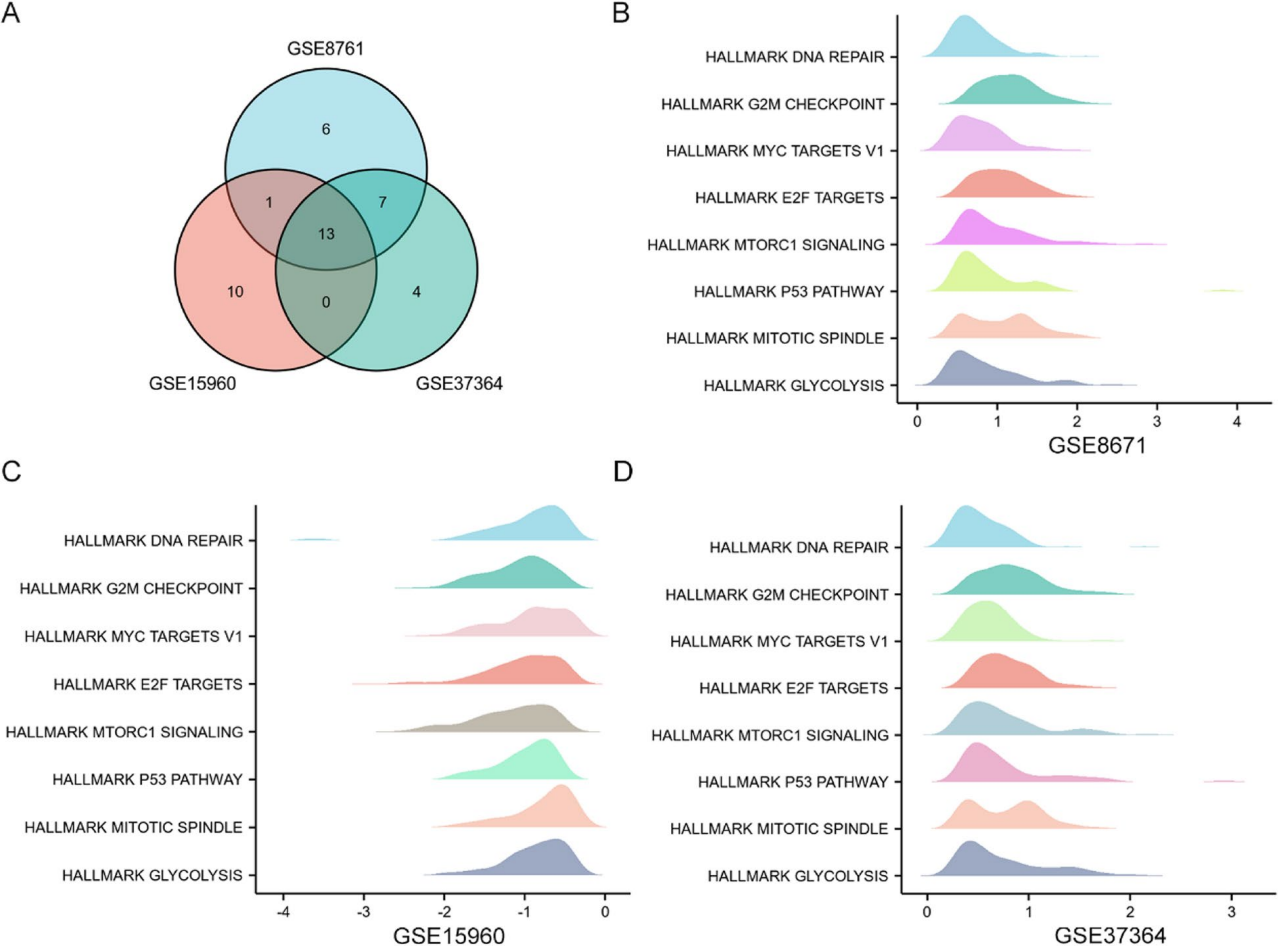
Gene set enrichment analysis (GSEA) of three GEO datasets. (**A**) Venn diagrams of GSEA among the three GEO datasets, including 13 common enriched pathways; (**B**) GSEA results of GSE8671; (**C**) GSEA results of GSE15960; (**D**) GSEA results of GSE337364. *p*-value < 0.05 and *p*-adjust < 0.05

Firstly, GO and KEGG pathway enrichment analyses were carried out to determine the molecular function of the common DEGs (Tables 2, 3 and 4). In up-regulated DEGs, KEGG analysis indicated that the Wnt signaling pathway played a role in CRA formation, and GO analysis revealed the main terms included growth factor activity, extracellular structure organization, neutrophil activation, and inflammatory response (Fig. 3A and B). KEGG analysis showed nitrogen metabolism and tryptophan metabolism enriched in the down-regulated DEGs (Fig. 3C). Similarly, GO analysis displayed that the main terms included carbonate dehydratase activity and bicarbonate transport (Fig. 3D). Nextly, GSEA was applied to the predicted biological function of three datasets. Venn plots displayed that the same functional pathways were enriched among the three databases(Fig. 4A). The analysis results showed that DNA repair, E2F, MYC, mTORC1, glycolysis, and mitotic spindle were dramatically enriched in CRAs (Fig. 4B-D).

**Table 2:** KEGG pathway analysis of DEGs related with CRA

**ONTOLOGY**	**ID**	**Description**	***P* value**	**p.adjust**	**geneID**	**Count**
Upregulated DEGs						
KEGG	hsa04310	Wnt signaling pathway	0.00033949	0.04854661	MMP7/PLCB4/AXIN2/LGR5/PLCB1/NKD1	6
KEGG	hsa04918	Thyroid hormone synthesis	0.00098001	0.07007047	PLCB4/PLCB1/DUOX2/DUOXA2	4
Downregulated DEGs						
KEGG	hsa00910	Nitrogen metabolism	0.0001264	0.01579962	CA1/CA2/CA7	3
KEGG	hsa00140	Steroid hormone biosynthesis	0.00044716	0.0279477	HSD11B2/HSD17B2/UGT2B15/DHRS11	4
KEGG	hsa00380	Tryptophan metabolism	0.00192267	0.08011124	ACAT1/MAOA/TPH1	3

**Table 3:** GO pathway analysis of upregulated DEGs related with CRA

ONTOLOGY	ID	Description	*p* value	p.adjust	geneID	Count
BP	GO:0,061,844	antimicrobial humoral immune response mediated by antimicrobial peptide	1.8644E-07	0.000194	DEFA5/DEFA6/REG3A/KLK7/REG1A/REG1B/CXCL11	7
BP	GO:0,002,283	neutrophil activation involved in immune response	2.1103E-05	0.00529344	SERPINA3/ANXA3/CD44/CHI3L1/CD55/LCN2/SERPINA1/S100A8/S100P/TCN1/OLFM4/QPCT	12
BP	GO:0,042,119	neutrophil activation	2.5754E-05	0.00529344	SERPINA3/ANXA3/CD44/CHI3L1/CD55/LCN2/SERPINA1/S100A8/S100P/TCN1/OLFM4/QPCT	12
BP	GO:0,043,062	extracellular structure organization	2.7981E-05	0.00529344	CD44/COL9A3/HYAL1/LPL/MMP7/MMP12/TNFRSF11B/SERPINB5/KLK7/TGFBI/TIMP1	11
BP	GO:0,006,959	humoral immune response	3.5213E-05	0.00563673	CD55/DEFA5/DEFA6/LCN2/REG3A/KLK7/REG1A/REG1B/S100A8/CXCL11	10
BP	GO:0,050,673	epithelial cell proliferation	0.00018119	0.01561372	BMP4/CDH3/ECM1/HYAL1/MMP12/REG3A/SERPINB5/REG1A/LGR5/SDR16C5	10
CC	GO:0,034,774	secretory granule lumen	2.0346E-08	2.1909E-06	SERPINA3/CHI3L1/DEFA5/ECM1/LCN2/PCSK1/SERPINA1/S100A8/S100P/TCN1/TIMP1/OLFM4/QPCT	13
CC	GO:0,060,205	cytoplasmic vesicle lumen	3.7348E-08	2.1909E-06	SERPINA3/CHI3L1/DEFA5/ECM1/LCN2/PCSK1/SERPINA1/S100A8/S100P/TCN1/TIMP1/OLFM4/QPCT	13
CC	GO:0,031,983	vesicle lumen	3.8664E-08	2.1909E-06	SERPINA3/CHI3L1/DEFA5/ECM1/LCN2/PCSK1/SERPINA1/S100A8/S100P/TCN1/TIMP1/OLFM4/QPCT	13
CC	GO:0,035,580	specific granule lumen	2.1678E-05	0.00092129	CHI3L1/LCN2/TCN1/OLFM4/QPCT	5
CC	GO:0,042,581	specific granule	0.00023912	0.00813023	ANXA3/CHI3L1/LCN2/TCN1/OLFM4/QPCT	6
CC	GO:0,062,023	collagen-containing extracellular matrix	0.00036907	0.01045705	SERPINA3/COL9A3/ECM1/SERPINA1/SERPINE2/S100A8/TGFBI/TIMP1/SBSPON	9
MF	GO:0,005,539	glycosaminoglycan binding	8.0044E-09	2.1052E-06	BMP4/CD44/LPL/MMP7/REG3A/SERPINE2/REG1A/REG1B/CXCL11/FGFRL1/CEMIP/REG4	12
MF	GO:0,008,201	heparin binding	5.515E-05	0.00705108	BMP4/LPL/MMP7/SERPINE2/CXCL11/FGFRL1/REG4	7
MF	GO:0,070,492	oligosaccharide binding	8.0431E-05	0.00705108	REG3A/REG1A/REG1B	3
MF	GO:0,005,201	extracellular matrix structural constituent	0.00253168	0.0443887	CHI3L1/COL9A3/ECM1/TGFBI/SBSPON	5
MF	GO:0,008,083	growth factor activity	0.00253168	0.0443887	BMP4/REG1A/TDGF1/TDGF1P3/TIMP1	5
MF	GO:0,001,227	DNA-binding transcription repressor activity, RNA polymerase II-specific	0.0027753	0.04561902	ASCL2/DACH1/MSX2/TBX3/NFE2L3/FOXQ1	6

**Table 4:** GO pathway analysis of downregulated DEGs related with CRA

ONTOLOGY	ID	Description	*p* value	p.adjust	geneID	Count
BP	GO:0,015,701	bicarbonate transport	4.7021E-05	0.03487239	CA1/CA2/CA7/SLC4A4	4
BP	GO:0,097,529	myeloid leukocyte migration	6.5068E-05	0.03487239	CHGA/IL6R/MST1/PIK3CG/SCG2/MST1L/MCOLN2	7
BP	GO:0,033,599	regulation of mammary gland epithelial cell proliferation	6.7845E-05	0.03487239	HOXA5/MST1/MST1L	3
BP	GO:0,007,585	respiratory gaseous exchange	0.00021793	0.08401364	HOXA5/NDN/SFTPA1/SFTPA2	4
BP	GO:0,033,598	mammary gland epithelial cell proliferation	0.00031469	0.09704933	HOXA5/MST1/MST1L	3
MF	GO:0,004,089	carbonate dehydratase activity	5.1772E-05	0.01304658	CA1/CA2/CA7	3
MF	GO:0,005,179	hormone activity	0.00035585	0.03184266	CHGB/GCG/PYY/SST/INSL5	5
MF	GO:0,033,764	steroid dehydrogenase activity, acting on the CH-OH group of donors, NAD or NADP as acceptor	0.00039536	0.03184266	HSD11B2/HSD17B2/DHRS11	3
MF	GO:0,052,689	carboxylic ester hydrolase activity	0.0005852	0.03184266	CA1/CA2/PNLIPRP2/CES2/PRDX6	5
MF	GO:0,016,229	steroid dehydrogenase activity	0.00069307	0.03184266	HSD11B2/HSD17B2/DHRS11	3
MF	GO:0,015,293	symporter activity	0.00075816	0.03184266	SLC22A5/SLC4A4/SLC13A2/SLC36A1/SLC16A9	5
MF	GO:0,008,194	UDP-glycosyltransferase activity	0.00093858	0.03378902	GCNT2/UGT2B15/B3GALT5/MGAT4A/B3GNT7	5

### Constructing PPI network and module analysis

**Fig. 5:**
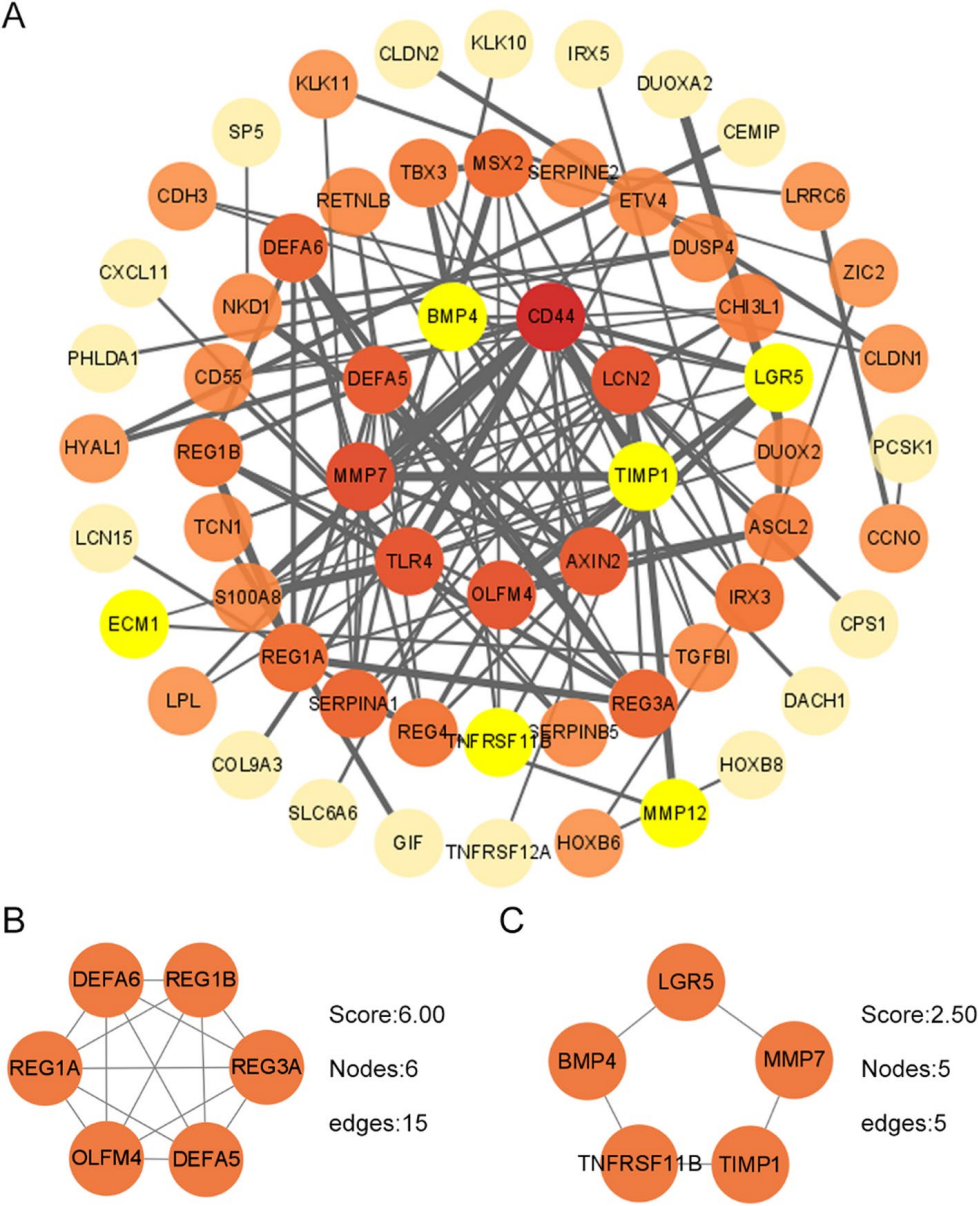
Establishment of PPI network and module analysis based on upregulated genes. **A** The whole PPI network of upregulated genes. **B-C** PPI networks of top three modules in upregulated DEGs through MCODE arithmetic. The top1 includes 6 nodes (DEFA5/6, REG1A/1B/3A, and OLFM4) and 15 edges. The top2 contains 5 nodes (LGR5, BMP4, MMP7, TIMP1, and TNFRSF11B) and 5 edges

**Fig. 6:**
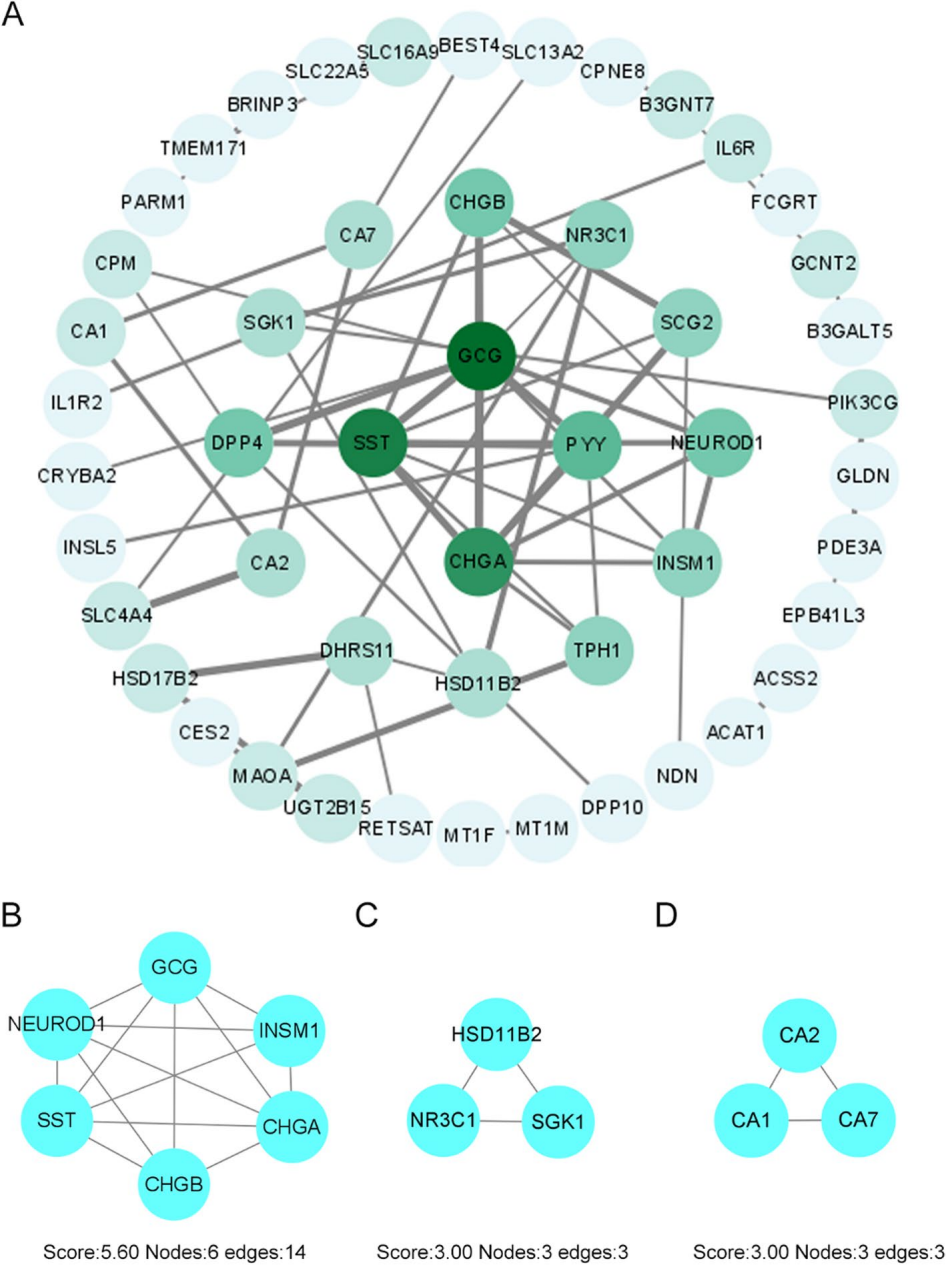
Performance of PPI network and module analysis based on downregulated genes. **A** The whole PPI network of downregulated genes. **B-D** PPI networks of top three modules in downregulated DEGs through MCODE arithmetic. The top1 includes 6 nodes (GCG, CHGB/A, SST, INSM1, and NEUROD1) and 14 edges. The top2 contains 3 nodes (NR3C1, SGK1, and HSD11B2) and 3 edges. The top3 consists of 3 nodes (CA1/2/7) and 3 edges

The STRING database was applied to create PPI networks to further explore the interactions between these DEGs. The top 3 modules were recognized from the upregulated PPI network by employing MCODE (Fig. 5A). The cluster 1 module included 6 nodes and 15 edges, the cluster 2 module included 5 nodes (Fig. 5B-C). The top 3 modules were identified from the upregulated PPI network (Fig. 6A). The cluster 1 module included 6 nodes and 14 edges; clusters 2 and 3 had 3 nodes and 3 edges, respectively (Fig. 6B-D).

As we know, the progression of CRC follows the mucosa-adenoma-adenocarcinoma sequence. Whether the genes are upregulated or downregulated in adenomas could persist in adenocarcinoma. We analyzed GEO datasets containing normal mucosa, adenoma, and adenocarcinoma (GSE37364 and GSE71187). The results showed that criticalgenes (REG1A and TIMP1) continued to increase. However, HSD11B2 and CA2 were reduced gradually (Supplementary Fig. 1).

### Exploring the correction of hub genes and stem-related genes

**Fig. 7:**
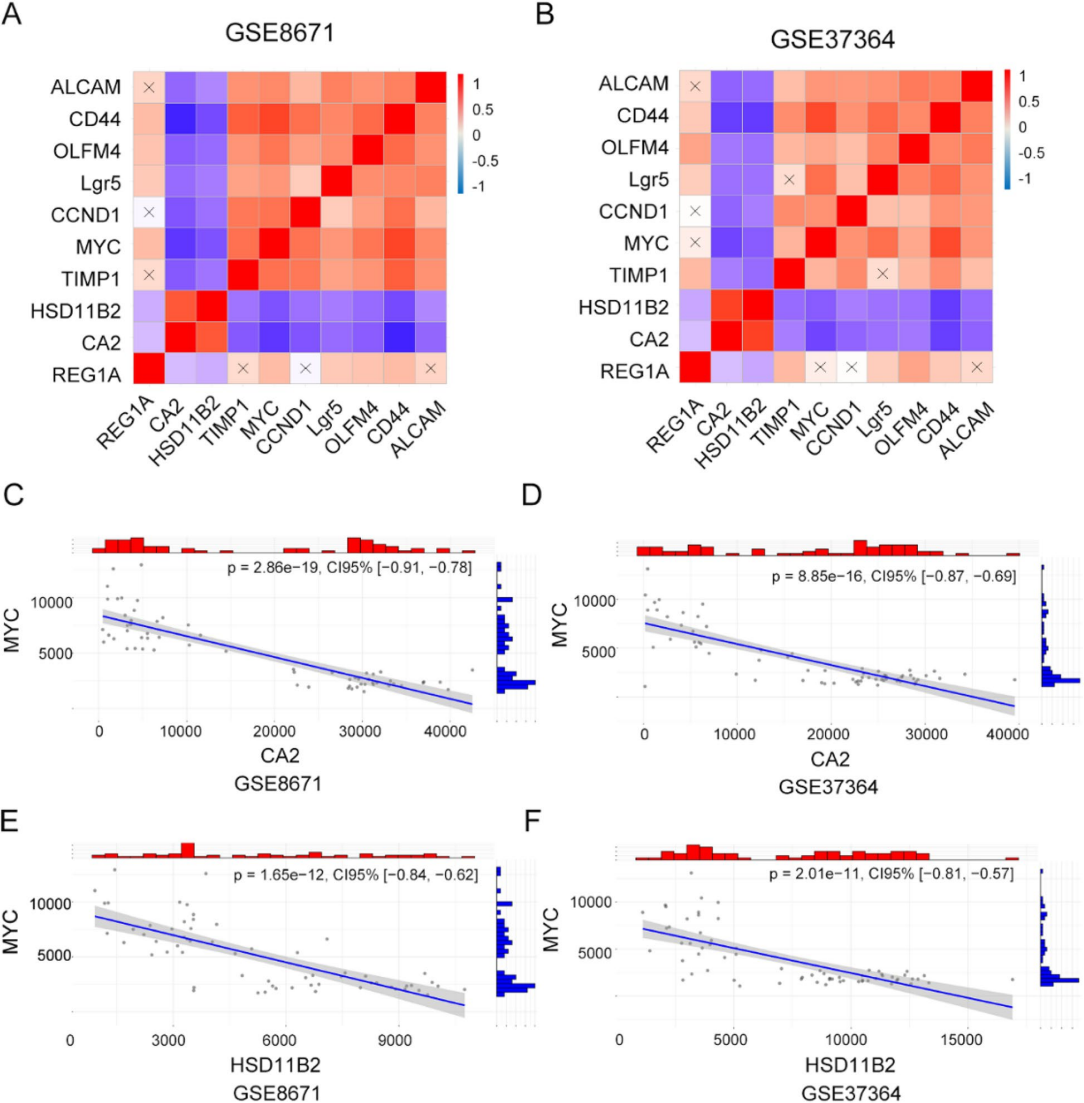
Association of hub genes and stem-related genes in GSE8671 and GSE37364. **A-B** The global relationship of hub genes and stem genes. X indicated *P*-value > 0.5. Red, positive relation; blue, negative relation. (**C-F**) The relationship of hub genes and MYC in GSE8671 and GSE37364

APC mutation in the intestine played an essential role in the progression of CRA [21]. Wnt/β-catenin is closely related to stemness and promotes tumor proliferation via regulation of stem genes [22]. KEGG analysis of up-regulated genes showed Wnt/β-catenin had already enriched in CRA. The downstream genes of β-catenin include Lgr5, MYC, and CCND1. So, in GEO datasets, we verified the association of hub genes and stemness-related genes (Lgr5, MYC, CCND1, CD44, Olfm4, and ALCAM). TIMP1 was positively associated with the stemness-related genes (*p*-value < 0.05), while CA2 and HSD11B2 had opposite trends (*p*-value < 0.05) (Fig. 7A and B). For example, TIMP1 was positively associated with MYC, while HSD11B2 and CA2 were negative (Fig. 7C-F). Besides, TIMP1 was positively associated with CCND1 and Olfm4, CA2 and HSD11B2 were negatively related with CCND1 and Olfm4 in GSE8761and GSE37364 (Supplementary Fig. 2). However, REG1A was not related to part of stem genes in GSE8671 and GSE37364.

### Validation of differentially expressed levels for hub genes

**Fig. 8:**
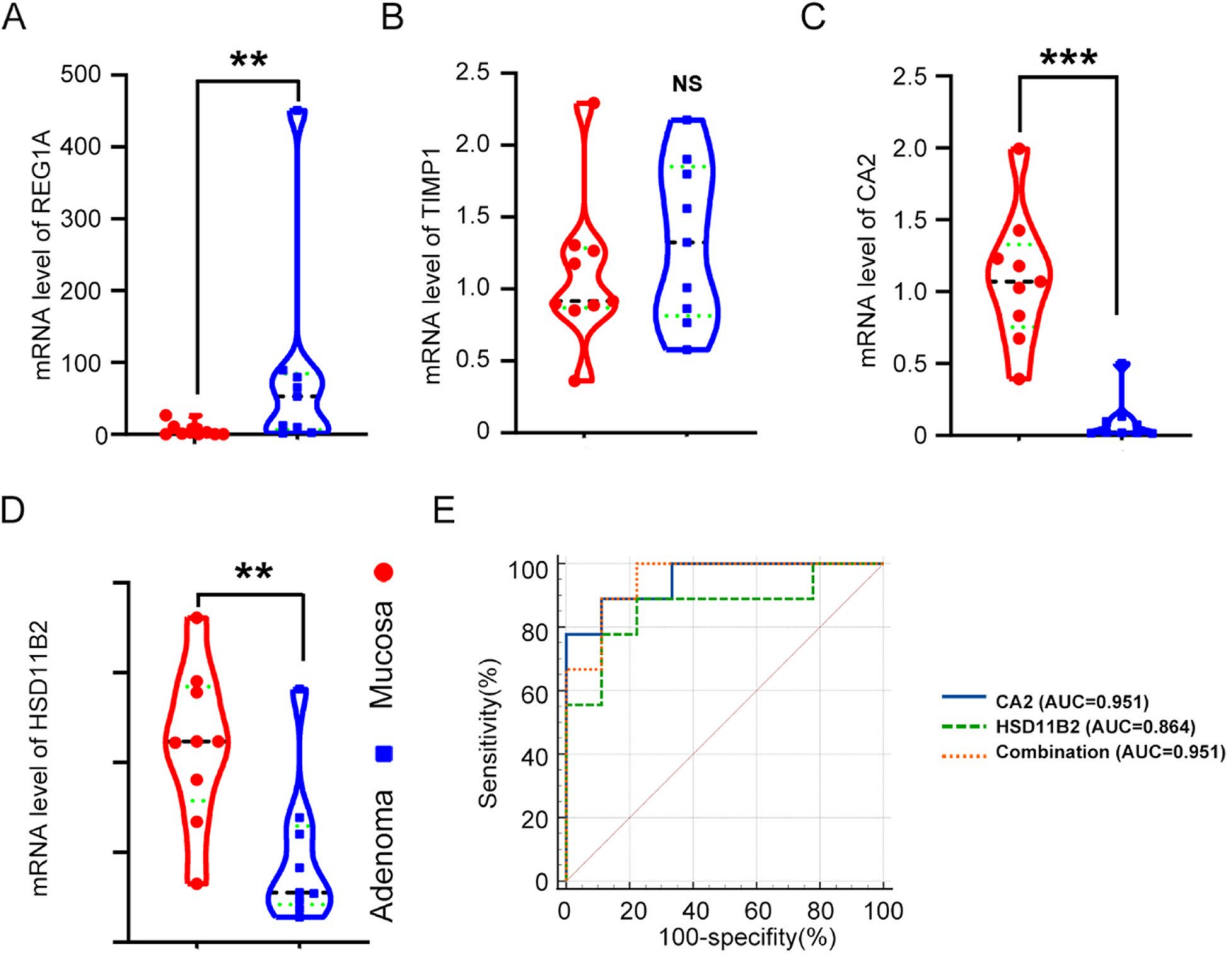
Screening and verification of the hub genes by RT-qPCR. **A-D** The relative expression of REG1A, CA2 and HSD11B2 between colorectal mucosa and adenoma were measured by RT-qPCR (*p*-value < 0.01). TIMP1 had no significant difference. **E** ROC curve with corresponding AUC value for hub genes when classifying CRA from mucosa. *** indicates *p-*value < 0.001, ** indicates *p-*value < 0.01, NS, none significance

**Fig. 9:**
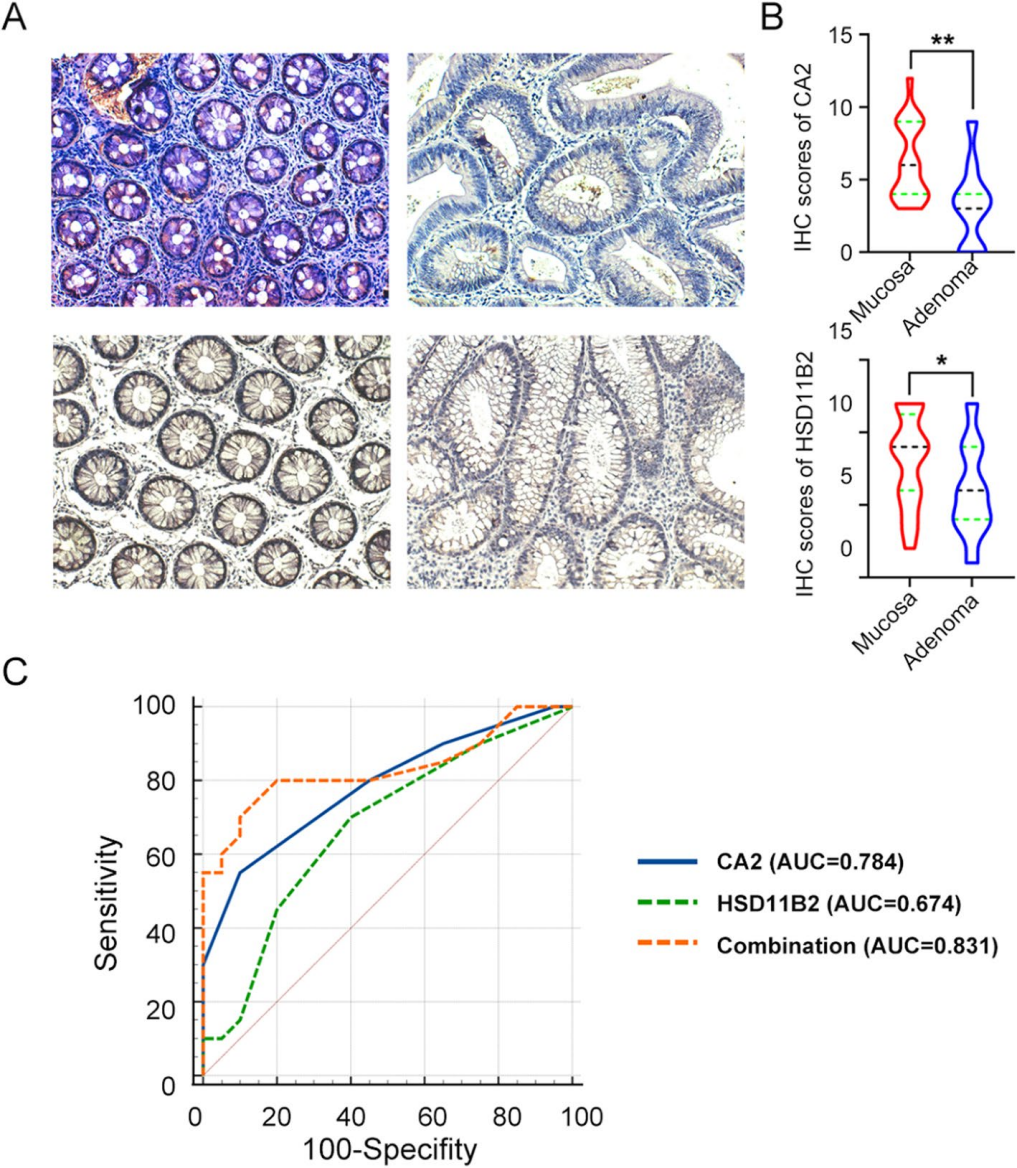
Confirming the expression of CA2 and HSD11B2 by IHC. **A** IHC of CA2 and HSD11B2 between colorectal mucosa and adenoma. **B** IHC scores of CA2 and HSD11B2. **C** ROC curve with corresponding AUC were valued by MedCalc based on the IHC scores of hub genes. *** indicates *p-*value < 0.001, ** indicates *p-*value < 0.01, * indicate *p-*value < 0.05; Scale bar, 200 μm

To verify the difference between these hub genes between adenoma and mucosa, we conducted RT-qPCR and immunohistochemical staining. RT-qPCR displayed that the mRNA expression level of hub genes was higher in the adenoma than in normal mucosa except TIMP1 (Fig. 8A-D). Combined with the above experimental results, we identified CA2 and HSD11B2 as core genes. Then, the ROC and AUC were applied to predict the diagnostic value of CA2 and HSD11B2 in distinguishing CRA from mucosa and CRC from CRA. The area under the curve (AUC) values of hub genes (CA2 and HSD11B2) and combination were 0.951, 0.864, and 0.951, respectively (Fig. 8E). In GSE41657and GSE71187, the ROC and AUC of hub genes could remarkably distinguish adenoma from mucosa or cancer from adenoma (Supplementary Fig. 3). During the sequence of mucosa-adenoma-carcinoma, the hub genes expression increased gradually and showed great significance. Next, we performed IHC to validate the protein levels of hub genes between mucosa and CRA. Consistent with the trend in the mRNA, the HSD11B2 and CA2 protein levels were reduced significantly (Fig. 9A-B). The area under the curve (AUC) values of CA2 and HSD11B2 and combination were 0.784, 0.674, and 0.831 according to IHC scores, respectively (Fig. 9C).

### Exploration of immune landscape of CRA GEO datasets

**Fig. 10:**
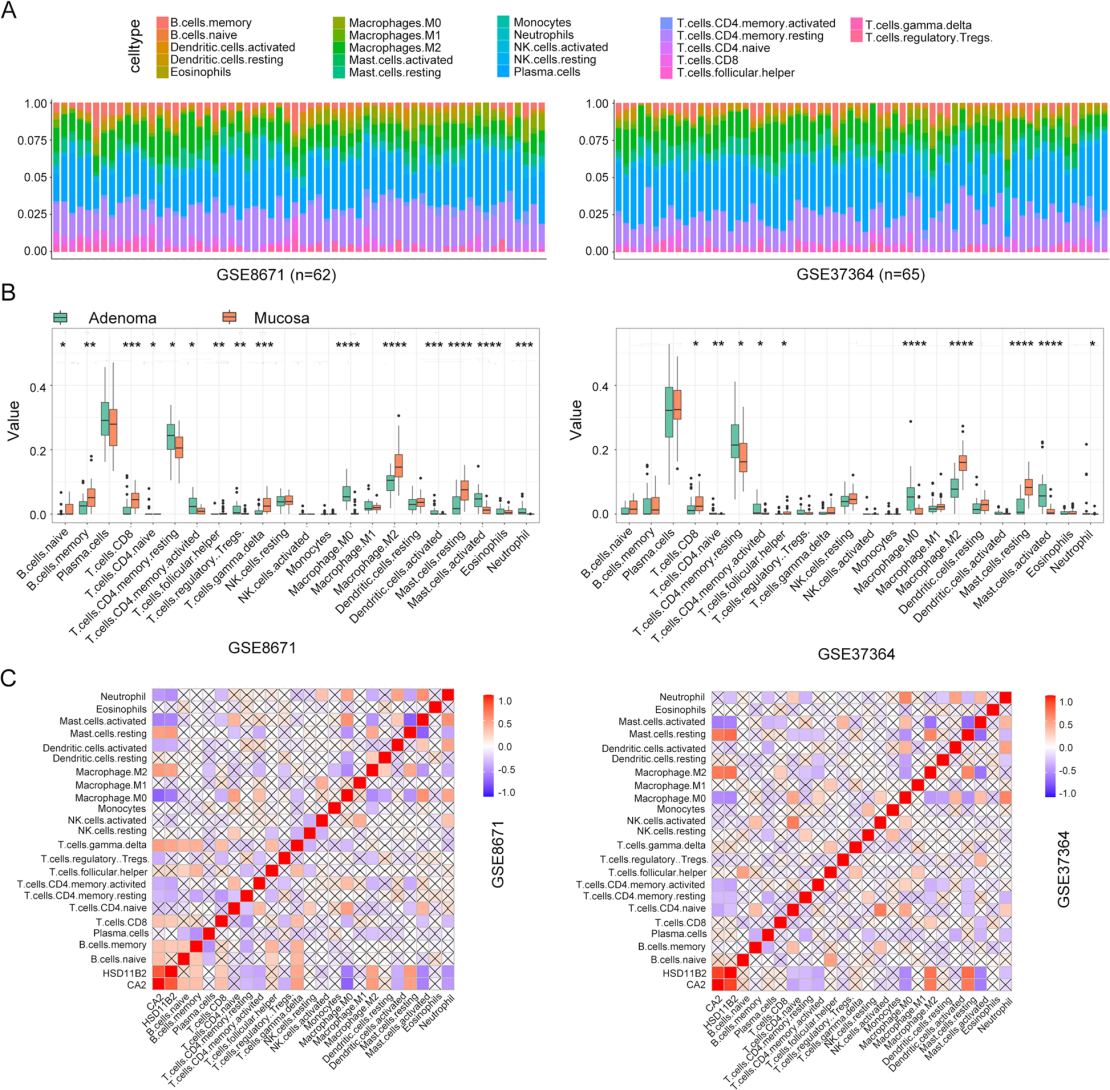
Relationship between CA2 and HSD11B2 expression and immune cell infiltration in CRA. **A** The 22 immune cells infiltrated among different tissues in GSE8671 and GSE37364. **B** The comparisons of 22 infiltrated immune cells between colorectal mucosa and adenoma. **C** The relationship of core genes (CA2 and HSD11B2) and 22 immune cell types

Tumor-infiltrating immune cells analysis was conducted by CIBERSORT in GSE8671 and GSE37364. Figure 10A displays the percentage of 24 immune cells infiltration in each sample. Infiltrating immune cells showed different infiltration abundance in adenoma or mucosa. We found the same infiltration trend of immune cells when comparing the two datasets (Fig. 10B). Naïve CD4 T cells, activated/resting memory CD4 T cells, macrophage M0, activated mast cells, and neutrophils were highly infiltrating in adenoma. Besides, Treg cells were enriched in adenoma in GSE8671, while it was not significant in GSE37364. However, CD8 T cells, follicular helper T cells, resting mast cells, and macrophage M2 highly infiltrated the mucosa. In the two GEO datasets, CA2 and HSD11B2 were negatively associated with neutrophils, activated mast cells and macrophage M0, positively with resting mast cells and M2 (Fig. 10C). These hub genes may activate neutrophils, mast cells and macrophage M0 and produce different inflammatory factors to promote the proliferation of adenoma epithelium.

### Risk score and survival analysis of CA2 and HSD11B2

**Fig. 11:**
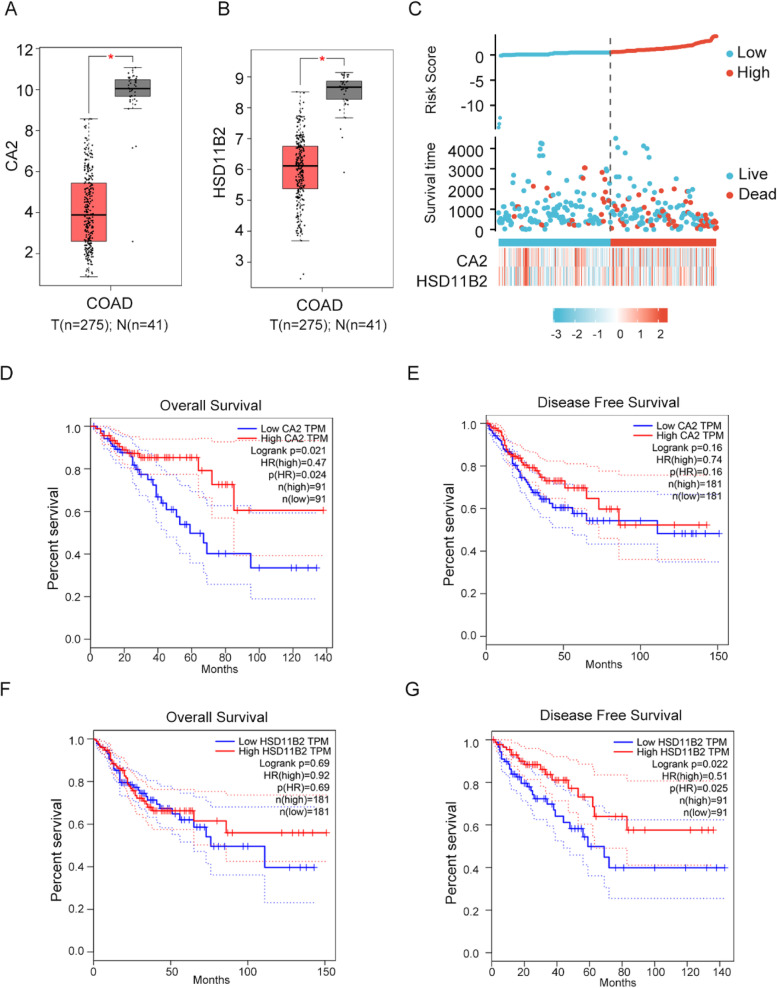
Risk score analysis and Kaplan–Meier (KM) survival curves for COAD with high and low CA2 and HSD11B2 mRNA expression in GEPIA. **A-B** CA2 and HSD11B2 expression levols in COAD according to TCGA database. T, tumor; N, normal. The number of tumors was 275, and the number of normal tissues was 41. **C** Construction of the core genes risk score analysis in the TCGA dataset (clinical characteristics of 330 patients) including risk score, survival time and gene expression. **D-E** KM survival curves for overall survival (*p*-value = 0.021) and disease free survival (*p*-value = 0.16) in COAD according to CA2 expression. (F-G) KM survival curves for overall survival (*p*-value = 0.69) and disease free survival (*p*-value = 0.022) in COAD according to HSD11B2 expression. COAD, colon adenocarcinoma. * indicate *p*-value < 0.05. HR: hazard ratios. The dotted line as the 95% CI

Finally, we explored the potential prognostic value of hub genes for CRC patients by risk score and Kaplan–Meier (KM) survival analysis and log-rank test. In COAD, CA2 and HSD11B2 were significantly decreased (Fig. 11A-B). To evaluate a robust risk signature for clinical use, we analyzed the risk score distribution, survival time, and core genes expression based on the TCGA dataset (Fig. 11C). Low-expressed CA2 and HSD11B2 were associated with poor prognosis. GEPIA was utilized to perform survival analysis based on colorectal adenocarcinoma samples from TCGA data. In the analysis of overall survival, high CA2 expression was related to prolonged OS (*p*-value = 0.024) (Fig. 11D), while HSD11B2 was not associated with OS (Fig. 11F). In the analysis of DFS (Disease-free survival), high HSD11B2 was related to prolonged DFS (*p*-value = 0.022) (Fig. 11G). Besides, CA2 was not associated with DFS (Fig. 11E). Finally, there was no relationship between hub genes and tumor stages in CRC (Supplementary Fig. 4).

## Discussion

Colorectal adenomas are precancerous lesions of colorectal cancer with high malignant potential, so the timely detection and diagnosis of adenomas are of great significance. At present, studies mainly focus on the discovery of CRC-specific biomarkers. However, colorectal adenomas are rarely reported, so finding hub genes that could drive the progression and deterioration of adenomas is more clinically meaningful for predicting high-risk adenomas. This study focused on colorectal mucosa and adenoma gene expression profiles via detailed bioinformatic analysis and uncovered significant regulatory signaling pathways and core genes.

Our study identified 230 robust DEGs by comparing genes expressed in colorectal mucosa and adenoma samples in three GEO datasets (GSE8671, GSE15960 and GSE37364), which included 127 upregulated and 103 downregulated genes. GO enrichment analysis of all genes indicated that growth factor activity, extracellular structure organization, neutrophil activation and inflammatory response were more potent in CRA samples than in mucosa samples. KEGG pathway that was enriched in CRA mainly included Wnt signaling pathway. Nitrogen and tryptophan metabolism was reduced in CRA. GO analysis revealed that growth factor activity, extracellular structure organization, neutrophil activation, and inflammatory response were enriched in CRA. Growth factor and extracellular structure organization can activate the cell proliferation signaling pathway and provide a sustainable 3D growing environment. Tumors can attract neutrophils to the cancer site via pro-inflammatory cytokine secretions and induce a switch to pro-tumoral (or N2) neutrophils, which support the metastatic spread and have an immunosuppressive role [23]. So, neutrophil activation and inflammatory response may contribute to excessive adenoma cell proliferation. GSEA showed that E2F, MYC, mTORC1, glycolysis and mitotic spindle were significantly enriched in CRA. E2F and MYC played a vital role in the proliferation of tumor cells [24, 25]. mTORC1 is involved in the metabolic regulation of tumors, and the upregulation of glycolysis is an essential feature of tumor metabolic reprogramming [26, 27]. The oncogenes and metabolic reprogramming may promote the progression of CRA. Next, the hub genes, screened by the PPI network and MCODE, were verified through the GEO datasets, including CA2 and HSD11B2. Combined with their expression level in CRA, CA2 and HSD11B2 were downregulated with statistical significance (*p*-value < 0.05). The relationship of stemness analysis suggested hub genes were negatively associated with tumor-stem genes. We also conducted tumor-infiltrating immune cells analysis by CIBERSORT in GSE8671 and GSE37364, and found different infiltration abundance in adenoma or mucosa. Hub genes were explored as the potential prognostic value for CRC patients by log-rank test and KM survival analysis. Therefore, according to our present research results, we hypothesized that CA2 and HSD11B2 might serve as biomarkers for the early diagnosis of CRA.

Carbonic anhydrase 2 (CA2) belongs to human carbonic anhydrases (CAs), a well-defined group of metal enzymes that catalyze carbon dioxide into bicarbonate [28]. CA2 functions to regulate ion transport and pH balance, which permeates many biological processes. CAs variants have been linked to ulcers, osteoporosis, obesity, and cancer [29]. The immunohistochemistry results in HCC revealed that CA2 expression levels were lower in tumor tissues than in adjacent tissues. The KM analysis demonstrated that DFS and OS were higher in the CA2 high expression group than in the CA2 low expression group (*p*-value < 0.05) [30]. Low CA2 expression is negatively correlated with cancer size, distant metastasis, pathological stage, and poorer overall survival in gastric cancer [31, 32]. The clinicopathological correlation analysis showed that CA2 was significantly downregulated in tumor metastases, such as hepatocellular carcinoma (*p*-value = 0.026) [33]. Low CA2 expression may promote adenoma cell stemness and serve as a biomarker for high-risk adenomas.

11-hydroxysteroid dehydrogenase (HSD11B2) is a catalytic enzyme that converts cortisol to cortisone and corticosterone to dehydrocorticosterone in vivo. HSD11B2, as a critical enzyme, can convert cortisol to inactive cortisone and accelerate tumor progression and metastasis [34]. Knockout of HSD11B2 promoted tumor angiogenesis (expression of EGFR and VEGFA), cell proliferation, and invasion in oral cancer cells [35]. HSD11B2 expression was significantly reduced in CRC tissues, which upregulated the expression of fibroblast growth factor binding protein 1 (Fgfbp1) and subsequently increased the phosphorylation of AKT to enhance cell migration and invasion [36]. HSD11B2 down-regulation in adenoma may promote its proliferation by promoting stemness and proinflammation.

Overall, in the study, we systematically explored the differences in molecular expression profiles of colorectal mucosa and adenomas, elucidating enriched pathways, hub genes (CA2 and HSD11B2), disease prognosis and immune patterns. However, our study had several limitations. First, more large clinical samples are needed to verify the expression of CA2 and HSD11B2. Moreover, the molecular functions of these hub genes in CRA remained unclear and needed to be verified. Using shRNA targeting these hub genes will further strengthen the reliability of this study.

## Conclusion

In conclusion, using various GEO datasets, we identified various significant DEGs in CRA, and found two hub genes that can be considered as novel and potential biomarkers of CRA. We further used the TCGA databases as a validation dataset to confirm the prognosis among CRC patients. Therefore, our research results present innovative and credible biomarkers for CRA, which will serve as a risk factor for predicting the malignant transformation of adenomas and be helpful for further clinical applications in CRA and CRC diagnosis, targeted therapy, and prognosis.

## Supplementary Information

**Additional file 1: Supplementary Figure 1.** The expression level of hub genes in GEO datasets. (A) The RPKM of hub genes (CA2, HSD11B2, TMIP1 and REG1A) in GSE37364. (B) The RPKM of hub genes (CA2, HSD11B2, TMIP1 and REG1A) in GSE71181. *** indicates *p* < 0.001, ** indicates *p* < 0.05, * indicate *p* < 0.05. **Supplementary Figure 2.** The association between hub genes (CA2 and HSD11B2) and stem-related genes (CCND1 and Olfm4) in GSE8671 and GSE37364. **Supplementary Figure 3.** ROC and AUC of hub genes among mucosa, adenoma and cancer in GEO datasets. (A) ROC curve with corresponding AUC value for hub genes when classifying CRA from the mucosa in GSE41657. (B) ROC curve with corresponding AUC value for hub genes when classifying CRA from CRC in GSE41657. (C) ROC curve with corresponding AUC value for hub genes when classifying CRA from the mucosa in GSE71187. (D) ROC curve with corresponding AUC value for hub genes when classifying CRA from CRC in GSE71187. CRC, colorectal cancer. **Supplementary Figure 4.** The relationship of hub genes (CA2 and HSD11B2) and CRC stages. **Supplementary Table 1.** Primers for RT-qPCR.

## Data Availability

The datasets support the findings of our study are available in GEO (Gene Expression Omnibus) repository, including https://www.ncbi.nlm.nih.gov/geo/query/acc.cgi?acc=GSE8671, https://www.ncbi.nlm.nih.gov/geo/query/acc.cgi?acc=GSE15960, and https://www.ncbi.nlm.nih.gov/geo/query/acc.cgi?acc=GSE37364. GEPIA database (http://gepia2.cancer-pku.cn/#index) was used to explore the relationship between gene expression and prognosis in CRC based on TCGA datasets.
