# Supplementary material for: Identification and clinical validation of key genes as the potential biomarkers in colorectal adenoma
Source: BMC Cancer. 2023 Jan 11;23:39. doi: 10.1186/s12885-022-10422-9 (PMC9832797; doi:10.1186/s12885-022-10422-9)
Supplement: Supplementary file 1 — Additional file 1: Supplementary Figure 1. The expression level of hub genes in GEO datasets. (A) The RPKM of hub genes (CA2, HSD11B2, TMIP1 and REG1A) in GSE37364. (B) The RPKM of hub genes (CA2, HSD11B2, TMIP1 and REG1A) in GSE71181. *** indicates p < 0.001, ** indicates p < 0.05, * indicate p < 0.05. Supplementary Figure 2. The association between hub genes (CA2 and HSD11B2) and stem-related genes (CCND1 and Olfm4) in GSE8671 and GSE37364. Supplementary Figure 3. ROC and AUC of hub genes among mucosa, adenoma and cancer in GEO datasets. (A) ROC curve with corresponding AUC value for hub genes when classifying CRA from the mucosa in GSE41657. (B) ROC curve with corresponding AUC value for hub genes when classifying CRA from CRC in GSE41657. (C) ROC curve with corresponding AUC value for hub genes when classifying CRA from the mucosa in GSE71187. (D) ROC curve with corresponding AUC value for hub genes when classifying CRA from CRC in GSE71187. CRC, colorectal cancer. Supplementary Figure 4. The relationship of hub genes (CA2 and HSD11B2) and CRC stages. Supplementary Table 1. Primers for RT-qPCR. [file 12885_2022_10422_MOESM1_ESM.docx]

***Supplement materials***

**Identification and Clinical Validation of Key Proteins as the Potential Biomarkers in Colorectal Adenoma**

# Supplementary Figures and Tables

**Supplementary Figures**


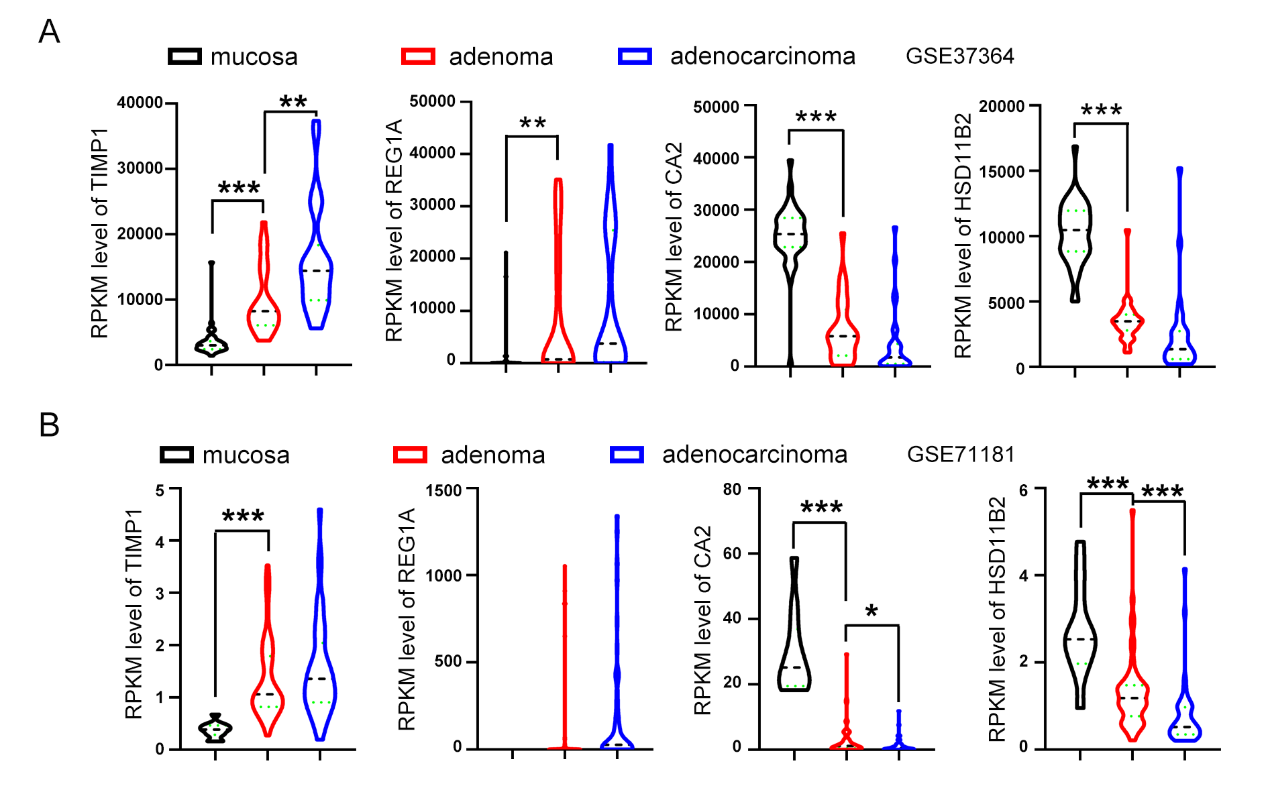


**Supplementary Figure 1. The expression level of hub genes in GEO datasets.** (A) The RPKM of hub genes (CA2, HSD11B2, TMIP1 and REG1A) in GSE37364. (B) The RPKM of hub genes (CA2, HSD11B2, TMIP1 and REG1A) in GSE71181. *** indicates p < 0.001, ** indicates p < 0.05, * indicate p < 0.05.


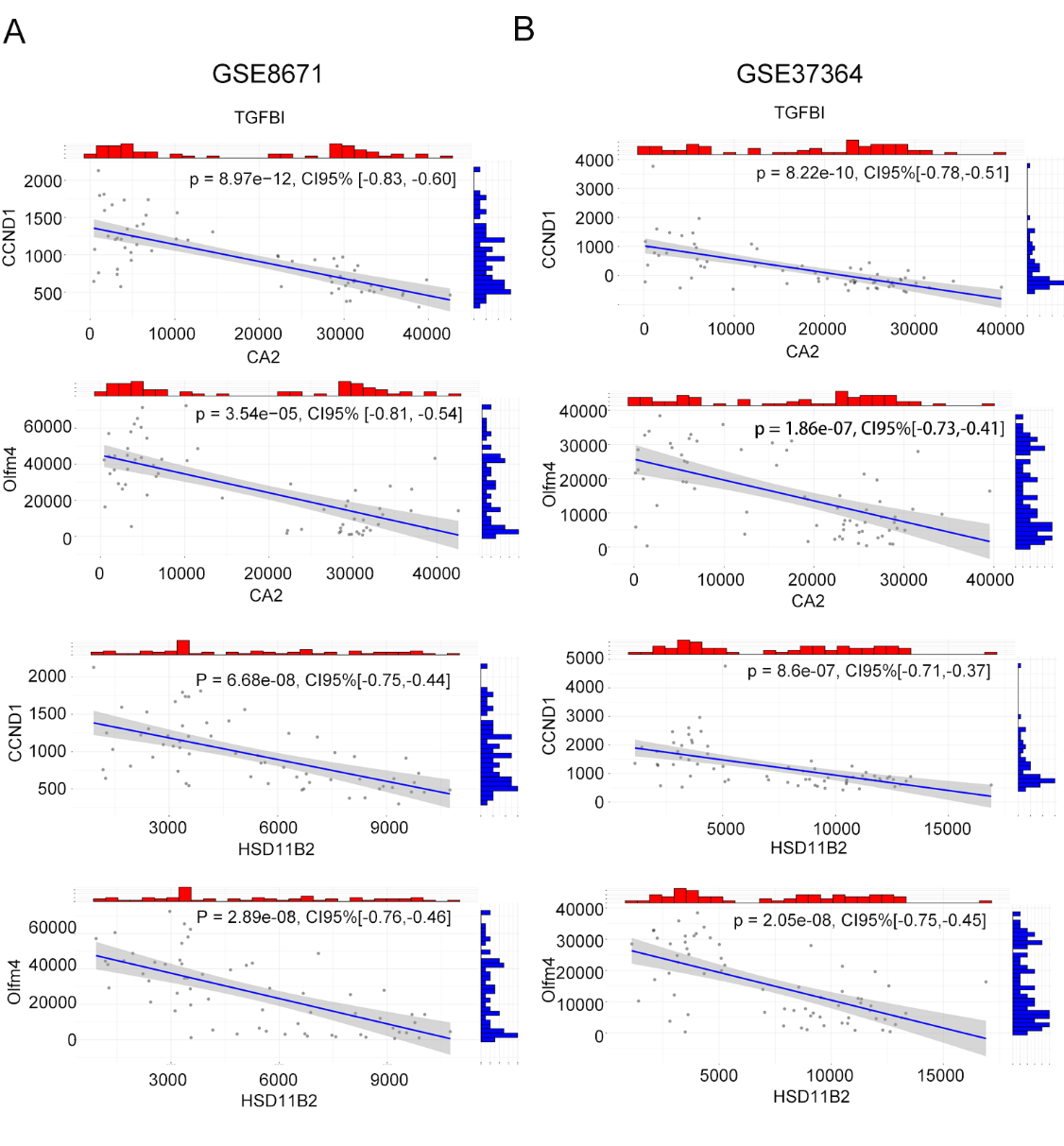


**Supplementary Figure 2. The association between** **hub genes (CA2 and HSD11B2) and stem-related genes (CCND1 and Olfm4) in GSE8671 and GSE37364.**


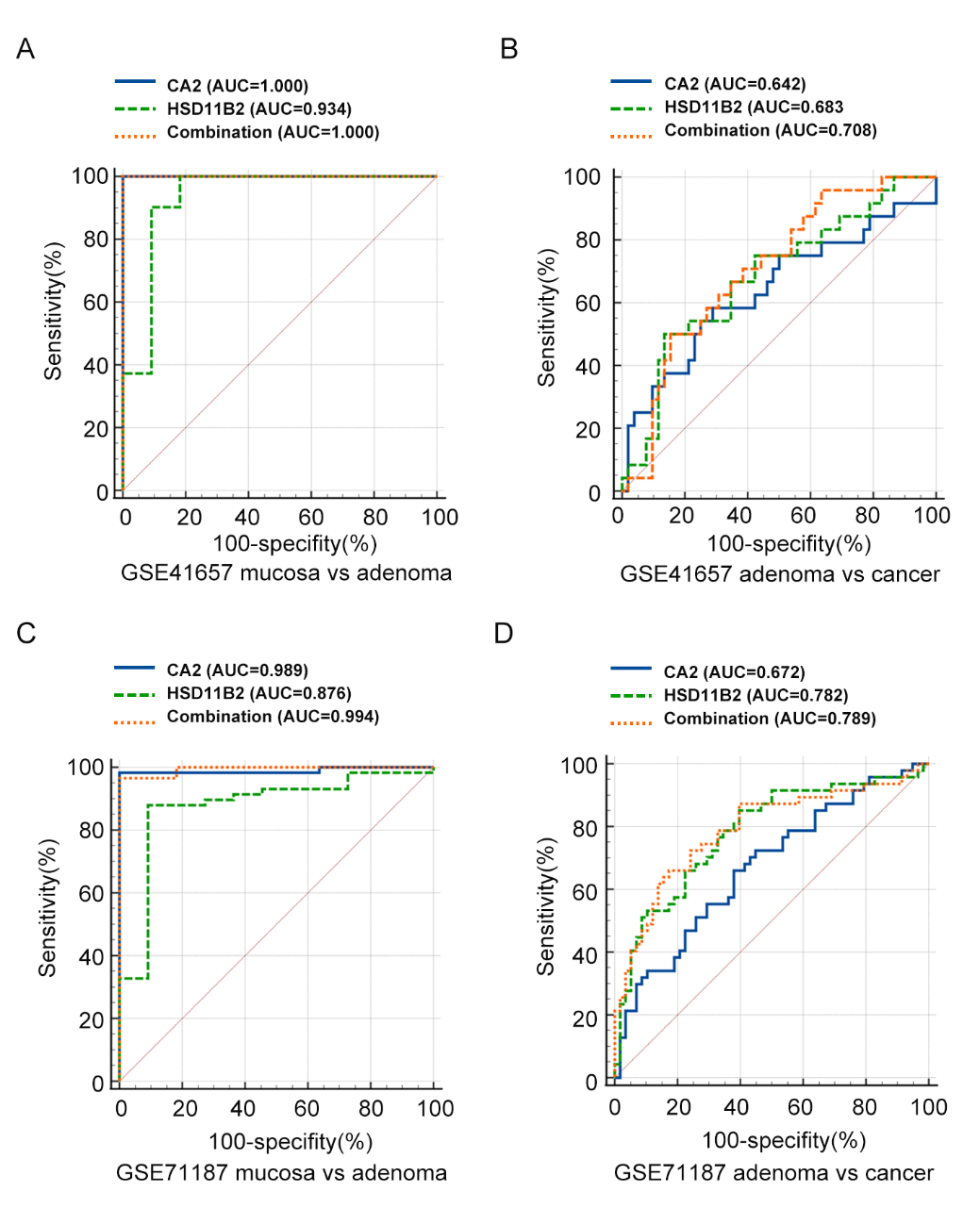


**Supplementary Figure 3. ROC and AUC of hub genes among mucosa, adenoma and cancer in GEO datasets.** (A) ROC curve with corresponding AUC value for hub genes when classifying CRA from the mucosa in GSE41657. (B) ROC curve with corresponding AUC value for hub genes when classifying CRA from CRC in GSE41657. (C) ROC curve with corresponding AUC value for hub genes when classifying CRA from the mucosa in GSE71187. (D) ROC curve with corresponding AUC value for hub genes when classifying CRA from CRC in GSE71187. CRC, colorectal cancer.


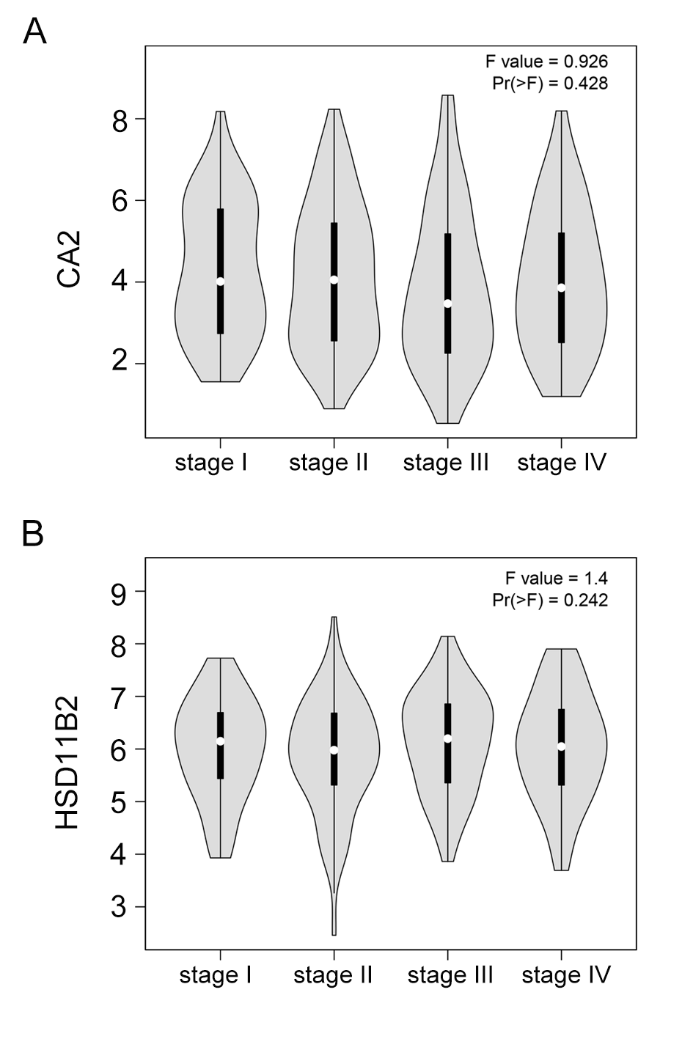


**Supplementary Figure 4. The relationship of hub genes (CA2 and HSD11B2) and CRC stages.**

1. **Supplementary Table**

**Supplementary Table 1. Primers for RT-qPCR.**

| REG1A-Forward | CCGGACCATCTCTCCAACTC |
| --- | --- |
| REG1A-Reverse | GGTTCCAAAGACTGGGGTAGG |
| TGFBI-Forward | ATGACCCTCACCTCTATGTACC |
| TGFBI-Reverse | CACAGTTCACAGTTACAATCCCA |
| TIMP1-Forward | CTTCTGCAATTCCGACCTCGT |
| TIMP1-Reverse | ACGCTGGTATAAGGTGGTCTG |
| HSD11B2-Forward | ATTAGCCGCGTGCTAGAGTTC |
| HSD11B2- Reverse | CCGCATCAGCAACTACTTCAT |
| CA2-Forward | ATCGACACTCATACAGCCAAGT |
| CA2-Reverse | AAAGCATGACCATTGTTGAGGA |
